# Pre-Harvest Strategy for Improving Harvest and Post-Harvest Performance of Kale and Chicory Baby Leaves

**DOI:** 10.3390/plants14060863

**Published:** 2025-03-10

**Authors:** Anna Bonasia, Corrado Lazzizera, Antonio Elia, Giulia Conversa

**Affiliations:** Department of Agriculture, Food, Natural Resources and Engineering (DAFNE), University of Foggia, 71100 Foggia, Italy; corrado.lazzizera@unifg.it (C.L.); antonio.elia@unifg.it (A.E.); giulia.conversa@unifg.it (G.C.)

**Keywords:** antioxidants, nitrate, clinoptilolite zeolite, methyl-jasmonate, elicitors

## Abstract

A greenhouse trial was conducted in Southern Italy to examine the effects of foliar applications of two substances, methyl-jasmonate (MeJA) and a zeolite, on the harvest and post-harvest performance of two hydroponically grown baby leaf genotypes (leafy chicory ‘Cicoria costa rossa’; kale ‘Cavolo nero’). MeJA is a phyto-hormone primarily studied for fruit and post-harvest applications, while zeolite is typically used for pest and disease biological control. MeJA (Sigma-Aldrich Merck KGaA, Darmstadt, Germany), and a commercial zeolite (Big-Zeo, Agricola Internazionale s.r.l., Pisa, Italy) (BigZeo) were sprayed twice at the second and fourth true leaf stages (BigZeo, 5 kg ha^−1^; MeJA, 250 µM). Bio-physiological (yield, dry matter DM, chlorophyll CHL, weight loss WL) and qualitative (nitrate, carotenoids, phenols, flavonoids, anthocyanins, antioxidant activity) traits were evaluated in both raw and fresh-cut (7 day-cold-stored) products. Treatments did not significantly affect yield (1.0 kg m^−2^), while plant responses to the substances concerning other traits were genotype-dependent. MeJA enhanced greenness (CHL), texture (DM), and antioxidant activity (by increasing carotenoids and flavonoids) in chicory. In contrast, zeolite improved greenness, texture, and antioxidant activity (by increasing carotenoids, anthocyanins, and phenols), and reduced nitrate in kale. Treatments did not affect weight loss (2.2 g 100 g^−1^ f.w., on average). After 7 days of storage, MeJA-treated chicory and zeolite-treated kale exhibited improved textural and nutritional quality.

## 1. Introduction

Baby leaf vegetables have become increasingly popular as a ready-to-eat salad mix of different immature leaves with attractive colors, shapes, and exceptional flavor and nutritional value. They are rich in bioactive compounds with potential health benefits, including anti-inflammatory, antioxidant, radical-scavenging, and gastro-protective properties. These compounds, including flavonoids, anthocyanins, vitamin C, and other phytochemicals, contribute to the overall health benefits of baby leaves [1]. However, baby leaves are highly perishable, posing a significant challenge for long-term storage [2]. Therefore, minimizing losses in terms of bio-physical quality, visual appeal, and nutrient content, both in pre- and post-harvest, is a crucial problem from an economic and environmental perspective [2].

Growing concerns about the use of synthetic substances for maintaining fresh produce quality have led to increased interest in the response of plant stress elicitors. These compounds play a vital role in various defensive processes, including the regulation of enzyme activity, cellular defense against oxidative damage, and abiotic stress tolerance [3].

Described in the 1970s, elicitors, primarily pathogen-derived compounds, were initially used to induce natural resistance in horticultural crops [3]. Today, elicitors are widely recognized as a preferred quality management tool, capable of enhancing phytochemical content (carotenoids, phenols, anthocyanins) in plants when applied (alone or in combination) at selected time points of vegetable growth [3].

While initially used to increase plant tolerance to pathogens, elicitors have been found to activate pathways involved in plant secondary metabolism. Plants respond to these substances by activating a range of defense mechanisms, like those triggered by pathogen infections or environmental stimuli. This ultimately affects plant metabolism and promotes the synthesis of phytochemicals.

Elicitors can be classified based on their origin: biotic, abiotic (chemical or physical), and phyto-hormones [3]. Among phyto-hormones, jasmonic acid, methyl-jasmonate (jasmonetes), methyl-salicylate, salicylic acid, ethylene, cytokinin, and gibberellin can be considered as elicitors [4]. Another important classification is the timing of elicitor application (pre-harvest, post-harvest, or a combination).

Methyl-jasmonate (MeJA) exhibits antibacterial properties and enhances disease resistance in fruits and vegetables by increasing antioxidant levels and defense-related enzymes [5]. MeJA has been shown to improve chilling tolerance, induce pathogen resistance, and enhance disease resistance, in order to maintain storage quality in various fruits and extend the shelf-life of fruits and vegetables [5,6].

MeJA is commonly used for fruits, primarily in post-harvest applications [7] and it is scarcely studied in vegetables. In particular, studies on vegetable pre-harvest application are limited to broccoli [8,9], chives [10], and coriander [11].

Zeolites are crystalline alumina-silicates characterized by a three-dimensional tetrahedral framework of SiO_4_ and AlO_4_ units. Owing to their unique physico-chemical properties, they have been extensively utilized as soil conditioners to mitigate various agronomic and environmental challenges, including soil and water pollution, heavy metal contamination, nutrient depletion, and inefficient water use efficiency issues in arid and semi-arid regions [12]. In addition to their role in soil, zeolites have been applied as foliar applications to enhance biological control of plant pests and diseases. This efficacy is attributed to their reversible dehydration capacity and the formation of protective particle films on leaf surfaces [13]. The formation of zeolite particle films has also been associated with a reduction in leaf temperature and transpiration rate and, under certain conditions, an enhancement in photosynthetic activity and gas exchange. Therefore, their use has been proposed for mitigating abiotic stress in crop [14,15,16], as well as serving as bio-stimulants to promote plant growth and resilience [13]. Furthermore, some studies suggests that zeolite applications can improve both the quality [14,15,16] and shelf-life of agricultural products [17]. While the majority of these studies have focused on fruit tree species, recent investigation has demonstrated positive effects on mineral nutrient uptake, silicon accumulation, heat tolerance and overall productivity in processing tomato crops [18]. Despite these promising findings, there remains a scarcity of scientific data regarding the pre-harvest application of zeolites in baby leaf crops and their potential impact on both harvest and post-harvest performance.

Considering these factors, this study aimed to investigate the efficacy of two substances used as ascertained/potential elicitors in improving the harvest and post-harvest performance of baby leaves. The selected elicitors were tested on two representative genotypes from the Asteraceae and Brassicaceae families, which are the most common botanical families commonly cultivated for baby leaves.

## 2. Results and Discussion

### 2.1. Raw Material

#### 2.1.1. Biophysical and Visual Traits of Baby Leaf Products at Harvest

At harvest, kale yielded higher than chicory (+14%), due to increased leaf number (+43%) and area (+20%) (Table 1). Kale leaves exhibited a darker green color, as indicated by instrumental (lower L and higher h°; Table 1) and analytical (higher chlorophyll pigments) determinations (Table 1) and were less vivid (lower C; Table 1) than chicory leaves.

**Figure 1:**
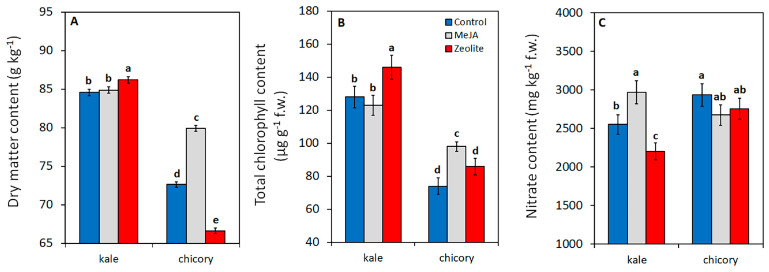
Effect of elicitors (methyl-jasmonate MeJA, zeolite, water-control)—on dry matter content (DM) (**A**), total chlorophyll content (CHL) (**B**), and nitrate content (**C**) in kale and chicory baby leaves, at harvest. Vertical bars (standard error) (DM, n = 9; CHL, n = 12; nitrate, n = 12;) with different letters are significantly different according to the LSD test (*p* = 0.05).

The application of elicitors did not affect the main productive parameters of the two baby leaf species. However, kale leaves exhibited higher dry matter (DM) and chlorophyll (CHL) content than chicory leaves when treated with zeolite compared to control and MeJA-treatments; on the contrary, in chicory the MeJA application significantly elicited DM and CHL levels compared to control and zeolite-treatments (Figure 1A,B).

Increased chlorophyll content is known to correlate with improved photosynthetic efficiency of the entire canopy, leading to higher dry yield and DM accumulation and/or fresh yield [19]. In our study, a relationship between CHL and DM accumulation was observed in specific genotype-elicitor combinations (Figure 1A,B), despite that this did not result in an increased fresh yield (Table 1).

Some studies have highlighted the growth-stimulating and productivity-enhancing properties of substances tested in this study, particularly in plant species under unfavorable growing conditions (e.g., drought, salinity), as seen for MeJA in rice [20] and in corn [21]. The same natural zeolite used in this work (micronized clinoptilolite) has been shown to enhance plant growth (as aboveground dry weight) and dry matter content of fruits in foliar-treated processing tomato, especially under stressful thermal conditions [18].

Zeolite treatment appeared to have a strong elicitation effect in kale, while depressing DM in chicory; conversely, chicory was responsive to MeJA (Figure 1A).

The physiological response of plants to the foliar zeolite applications appears to be directly linked to the structural characteristics of zeolite and the formation of a coating film on the leaf surface. Carter et al. [22] reported that particulate zeolite sprays could alter the optical properties of leaves, increasing foliage reflectivity and potentially reducing photosynthetic efficiency. Comparative studies of coating particle films have indicated that kaolin in comparison to CHA-zeolitite [14], and the Cuban zeolite in comparison to (micronized clinoptilolite) zeolite [18] exhibit a greater tendency to form continuous coating films. These properties are associated with modifications in leaf light reflectance, which may lead to decreased efficiency in photosynthetic performance [14,18].

**Figure 2:**
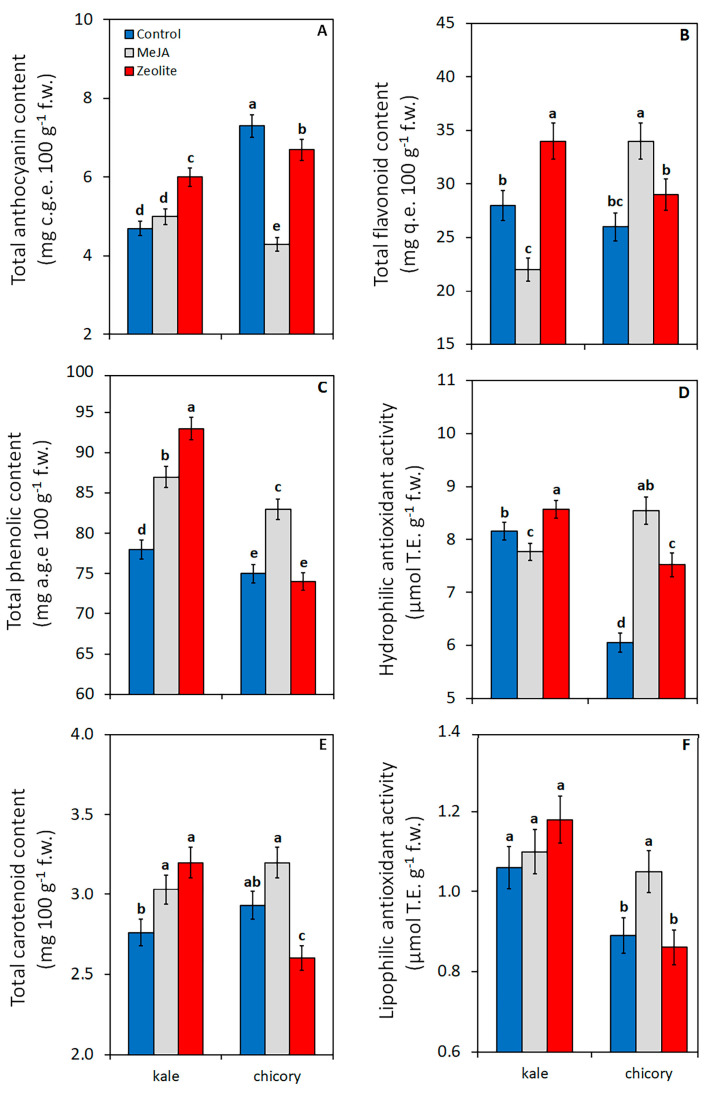
Effect of elicitors (methyl-jasmonate MeJA, zeolite, water-control) on content of total anthocyanins (**A**), flavonoids (**B**), phenolics (**C**), on hydrophilic antioxidant activity (**D**), on content of total carotenoids (**E**), and on lipophilic antioxidant activity (**F**) in kale and chicory baby leaves at harvest. Vertical bars (standard error) (n = 12) with different letters are significantly different according to the LSD test (*p* = 0.05).

In chicory, observed reductions in dry matter content (Figure 1A) and photoprotective pigments (carotenoids, Figure 2E) suggest the formation of a thicker and/or more continuous coating, resulting in increased spectral reflectance for this species. It is plausible that interspecies variation in leaf morphology and in the composition of cuticular waxes, particularly between members of Asteraceae and Brassicaceae families [23,24], influenced the characteristics of the particle films formed.

On the contrary, this response was not observed in kale, where zeolite application even enhanced dry matter accumulation and chlorophyll content, indicating a potential bio-stimulant effect. The role of zeolite coating films in generating bioavailable (soluble) forms of silicon, which act as bio-stimulants [25], has been previously demonstrated [16]. Although this study did not assess leaf silicon concentration, previous research has correlated higher silicon levels in fruits with enhanced plant growth in a processing tomato crop treated with (micronized clinoptilolite) zeolite [18]. Further investigation is needed to characterize the properties of zeolite particle films and elucidate their interactions with leaf morphological and anatomical structures.

Additionally, the specific leaf characteristics of kale may have mitigated interactions with MeJA, resulting in a limited physiological response.

#### 2.1.2. Antioxidant Compounds and Activity of Baby Leaf Product at Harvest

Baby leafy vegetables are recognized as excellent sources of antioxidant molecules, including phenolic compounds (such as anthocyanins and flavonoids), vitamin C, and carotenoids [26,27]. The overall antioxidant activity, reflecting the combined efficiency of all antioxidant compounds in scavenging free radicals, encompasses both hydrophilic (HA) (associated with anthocyanins, flavonoids, phenols, vitamin C, and others) and lipophilic (LA) components (more negligible, associated with carotenoids, and others, such as tocopherols and lipophilic phenols) [28].

##### Hydrophilic Component of Antioxidant Activity of Baby Leaf Product at Harvest

The relative data are reported in Table S1. Kale baby leaf treated with zeolite exhibited higher anthocyanin (Figure 2A), flavonoid (Figure 2B), and total phenolic (Figure 2C) content compared to the control and MeJA treatment, aligning closely with the hydrophilic component of antioxidant activity (HA) (Figure 2D). In chicory, zeolite treatment exhibited a negligible effect on these bioactive compounds (Figure 2A–C) despite increasing the HA (Figure 2D).

The MeJA application in kale also improved phenols, whereas it inhibited flavonoid accumulation (Figure 2B) and reduced the HA (Figure 2D). MeJA was more effective in enhancing the antioxidative proprieties of chicory by prompting the accumulation of flavonoids and phenols (Figure 2B,C), resulting in the highest value of HA for this species (Figure 2D).

The effects of endogenous/exogenous hormones, like jasmonic acid, on flavonoid accumulation have been extensively studied [29]. It is noteworthy that exogenous MeJA enhanced phenols in broccoli [8] and phenols along with flavonoids in tomato [30] by promoting enzymatic activities associated with the phenyl–propanoid pathway [30,31,32]. Moreover, exogenous MeJA (in combination with alanine) increased flavonoid content in *Populus wutunensis* leaves and seedlings, especially under salt stress [32].

In this study, in both species it can be confirmed the phenolic elicitation by MeJA, despite a genotype-dependent response, was observed as difference in accumulations of specific groups of phenols (flavonoids in kale and anthocyanins in chicory). A plausible hypothesis is that stressful conditions may be concurrently necessary to elicit flavonoid synthesis in kale leaves by MeJA.

Published data on the effects of zeolite foliar application on antioxidative compound levels in products are scarcer. Foliar pre-harvest application of zeolite in grape increased anthocyanin content in ‘Sangiovese’ berries [33]. A natural zeolite solution sprayed on table grape clusters 48 h before harvest improved the flavonoid content and antioxidant activity of fruit [17], proving that this substance can activate different secondary metabolism routes.

##### Lipophilic Component of Antioxidant Activity of Baby Leaf Product at Harvest

The relative data are reported in Table S1. Both elicitors in kale leaves exhibited higher carotenoid content compared to the control (Figure 2E) without affecting the lipophilic component of antioxidant activity (LA) (Figure 2F). In chicory, zeolite treatment inhibited carotenoid accumulation (Figure 2E) along with the LA (Figure 2F), while MeJA application contributed to promoting carotenoids (Figure 2E) and other lipophilic compounds, clearly increasing LA (Figure 2F).

Numerous studies have demonstrated significant increases in carotenoid levels due to exogenous MeJA application, including in maize sprouts [34], maize kernels [35], cut rose flowers [36], and broccoli [8]. As shown in a study of rosemary suspension cells [37], MeJA up-regulates the expression of key genes involved in the carotenoid biosynthetic pathway.

Given the photo-protective function of carotenoids [38], the reduction of these compounds in chicory treated with zeolite (Figure 2E) can be attributed to an increased leaf light reflectance.

#### 2.1.3. Nitrate in Baby Leaf Product at Harvest

Baby leaves are food products containing significant amounts of nitrate. As highlighted by European Commission regulations, setting maximum nitrate levels in various foods, including vegetables such as spinach, lettuce, and rocket salad (EU 915/2023), nitrate accumulation poses a health risk.

In our research, nitrate levels varied significantly across genotype–elicitor combinations (Figure 1C). While neither elicitor affected nitrate levels in chicory products, MeJA foliar application increased nitrate accumulation in kale leaves, whereas zeolite decreased it compared to the control (Figure 1C); thus, in kale, zeolite seems to prompt N/nitrate assimilation, while MeJA is less efficient.

There is limited research on the effects of the substances used as elicitors in the present study on nitrate accumulation, regarding N uptake and particularly N assimilation. Rossato et al. [39] demonstrated that MeJA, supplied through the nutrient solution in a floating system, inhibited N/nitrate uptake in leaves and taproots of oilseed rape (*Brassica napus* L.) as a senescence factor. Similarly, Janicka et al. [40] confirmed the inhibition of nitrate uptake in cucumber seedlings.

It is possible to assume that a lesser N assimilation efficiency by MeJA could be imputable to a decrease of NR activity (a substrate-induced enzyme, catalyzing the first step of nitrate assimilation), as seen in the roots of JA-treated cucumber seedlings [40]. The inhibition of NR activity is probably due to the reduction of JA-induced nitrate uptake [40].

Some research described the improvement of (micronized clinoptilolite) zeolite, applied as a soil amendment, on N uptake in grain [41], but up to now there is no evidence related to zeolite leaf application and N uptake/assimilation. A higher N assimilation by zeolite application could be strictly related to a more efficient photosynthetic process, due to a higher chlorophyll content (Figure 1C), as proved by higher dry mass content (Figure 1C).

Based on the classification proposed by Santamaria [42], our products (2681 mg kg^−1^ f.w., on average) fall between ‘high’ (1000–2500 mg kg^−1^ f.w.) and ‘very high’ (2500–5000 mg kg^−1^ f.w.) nitrate levels. However, their nitrate levels are considerably lower than the maximum nitrate levels set by EU 915/2023 for autumn leafy species.

### 2.2. Stored Product

#### 2.2.1. Headspace Gases and Weight Loss of Stored Product

Monitoring headspace gases, O_2_ and CO_2_, in the bags of stored leaves is useful for assessing product respiration activity. As shown in Table 2, a genotypic effect emerged: the headspace gas composition of packed kale baby leaves (higher CO_2_ and lower O_2_) indicated a higher respiration rate than stored chicory product (Table 2). However, these differences were negligible so that total, weight losses, including respiration, and transpiration components, were unaffected by both genotype and elicitor application, averaging 2.2 g per 100 g of fresh weight (Table 2).

#### 2.2.2. Visual Quality, Anti-Nutritional and Antioxidant Compounds of Stored Baby Leaf Product

**Figure 3:**
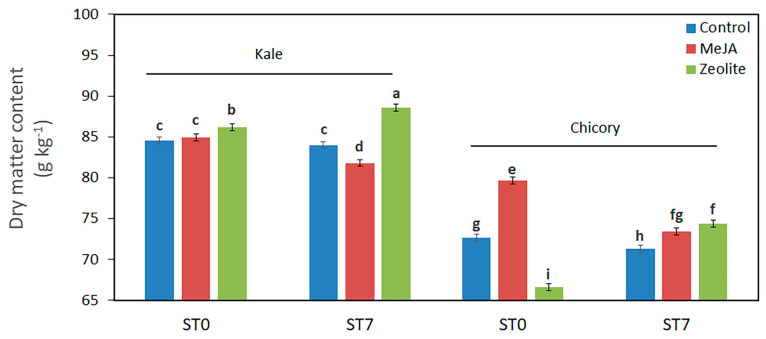
Effect of elicitors (methyl-jasmonate MeJA, zeolite, water-control) on dry matter content of kale and chicory baby leaves, at harvest (ST0) and after 7 days of storage (ST7). Vertical bars (standard error) (n = 9) with different letters are significantly different according to the LSD test (*p* = 0.05).

Dry matter (DM) accumulation, serving as an indicator of consistency, is a critical quality attribute of both fresh and fresh-cut baby leaves. During storage, DM content can be influenced by various factors, including pre-harvest agronomic practices and genotypic characteristics [43]. Zeolite-treated products exhibited an increase in DM content over the storage period. Specifically, kale treated with zeolite achieved the highest DM levels, reflecting superior consistency (Figure 3). This response aligns with findings from Huwei et al. [17], who reported similar effects in table grape berries. On the contrary, products treated with MeJA showed a decrease in DM content during storage (Figure 3). However, MeJA-treated chicory showed slightly higher DM levels compared to zeolite and control (Figure 3), suggesting an improvement in leaf texture.

Color is a primary external attribute influencing consumer purchase decisions [44]. During storage, variations in chlorophyll (decrease) and main color indices (increase in L*, decrease in h°, and increase in C*) indicated a loss of greenness (Table 3). Despite that the decrease in hue angle was more pronounced in kale (−9%) than in chicory (−4%) (Figure S1), at the end of the 7 days stored kale leaves exhibited a greener color compared to chicory product (Figure S1); this was probably due to the initial higher chlorophyll content (Table 1; Figure 1B).

Regarding the anti-nutritional profile after the storage period, the product showed a lower nitrate content, regardless of genotype or elicitor application (−19%; Table 3). While a potential increase in nitrites following a decrease in nitrates, due to cold and dark storage conditions, could be hypothesized, this trend remains controversial [45].

In terms of nutritional compounds, phenolic content was not affected by storage or pre-harvest treatments, averaging 77.5 mg a.g.e. 100 g^−1^ f.w. (Table 3). The hydrophilic (HA) of baby leaves, overlapping with the total antioxidant capacity (TAA), significantly increased during storage (+26%) (Table 3). Although a general decrease in flavonoids (−28%) and anthocyanins (−30%) occurred during storage, the overall increase in TAA(HA) is supported by the hypothesis that many antioxidant compounds are produced as secondary metabolites in response to stress (such as cold/storage) to compensate for its effects, like membrane damage [46]. Other hydrophilic compounds (like vitamin C), not determined in this research, might have contributed to the general increase in TAA(HA).

No interactions between storage/genotype/elicitor were observed for nitrate, antioxidant compounds, and antioxidant activity (Table 3). The effects of pre-harvest application of MeJA and zeolite in chicory and kale observed at harvest were maintained in the stored products. Thus, after 7 days of storage, the MeJA–chicory and zeolite–kale combinations exhibited a significant high-quality profile (appearance, nitrate, and antioxidant status).

## 3. Materials and Methods

The trial was conducted under autumn conditions in a plastic greenhouse at a commercial farm in Mola di Bari (BA), southern Italy (41°02′16.3″ N 17°06′20.3″ E, 5 m a.s.l.). The mean maximum and mean minimum temperatures of the cultivation period (5 November–12 December 2022) were 22.6 and 10.9 °C, respectively.

The kale (*Brassica oleracea* L., *Acephala* group; ‘Cavolo Nero’) (kale) and leafy chicory (*Cichorium intybus* L., *Foliosum* group; ‘Cicoria costa rossa’) (chicory) (De Corato Sementi, Andria, BT, Italy) were grown as baby leaf. Seeds were manually distributed on the substrate surface at a density of 1500 seeds m^−2^ in grow-box trays (23 × 48 × 6 cm, W × L × D) filled with peat (Brill 3 TYPical, Gebr. Brill Substrate GmbH & Co. KG, Georgsdorf, NI, Germany). During the first 4 days, the trays were placed in a germination chamber. On day five, seedlings were exposed to light inside the greenhouse.

Upon emergence, trays were arranged on aluminium benches (420 × 180 cm), and an ebb and flow irrigation system was adopted. Trays were irrigated daily with rainwater until germination was complete. After germination, a half-strength Hoagland nutrient solution was used. This was prepared by mixing macro- and micro-nutrients with rainwater, resulting in a final concentration (mg L^−1^) of 112 N, 120 K, 80 Ca, 31 P, 16 S, 12 Mg, 0.135 B, 0.56 Fe, 0.055 Mn, 0.0655 Zn, 0.016 Cu, and 0.025 Mo. A fertilizer dosing system (Priva NutriFit, De Lier, The Netherlands) was employed.

The applied substances were (a) methyl-jasmonate (MeJA), a phyto-hormone derived from jasmonic acid (Sigma-Aldrich Merck KGaA, Darmstadt, Germany), and (b) a commercial natural zeolite (Big-Zeo, 91% clinoptilolite, Agricola Internazionale s.r.l., Pisa, Italy).

The substances were carefully sprayed (500 L ha^−1^) twice during the cultivation cycle, at the 2nd and 4th true leaf stages, using a hand-sprayer. The untreated control received distilled water.

The framework of the experimental study is reported in Figure S2.

A randomized complete block design (RCBD) with three replications (benches) was used. The experimental unit consisted of 10 trays per treatment, totaling 180 trays. Trays within each bench were rotated daily to ensure uniform environmental conditions.

Plants were harvested 38 days after sowing by manually cutting the leaf bunches about 1 cm above the collar when they reached the optimal stage for fresh consumption as baby leaves. The harvested product was evaluated immediately (ST0-storage time 0) and after 7 days of dark-storage (ST7-storage time 7).

The storage was performed by packaging 150 g of raw material in OPP bags (20 μm) (Metalvuoto spa, Roncello, MI, Italy) (29.5 × 24.3 cm) in a refrigerator at 5 °C for 7 days.

### 3.1. Visual Traits, Biophysical and Chemical Determination of Raw Material

#### 3.1.1. Visual Traits and Biophysical Determination of Raw Material

Biophysical determinations conducted at harvest, including yield, fresh weight, number and leaf area, dry matter content, and color analyses, were performed on fresh material, considering three sub-samples for each replicate.

Leaf area was measured using a LI–COR 3100 (LICOR, Lincoln, NE, USA). Fresh plant material was first weighed to determine the fresh weight (FW). The samples were then dried in a thermo-ventilated oven at 70 °C until constant mass (DW).

Dry matter content (DM) was calculated as [(DW/FW) × 100]. Results were expressed on a fresh weight basis. Weight losses due to total transpiration, respiration, and other factors were calculated as described in Bonasia et al. [47].

Color measurements were acquired in the CIELAB (1976) color space parameters: L* (lightness index), a* (chroma component), and b* (chroma component). A portable tri-stimulus color meter (Minolta Chroma Meter CR-200; Minolta Camera Co., Ltd., Osaka, Japan) was used. Derived parameters, such as hue angle (h°) and chroma (C*), indicating hue and color intensity, were calculated.

#### 3.1.2. Chemical Determinations of Raw Material

Harvest determinations encompassed chemical determinations, as chlorophyll, nitrate, cations, phenols, flavonoids, anthocyanins, carotenoids, and antioxidant activity, on lyophilized samples (ScanVac, Chemie s.r.l., Valenzano, Bari, Italy), ground using a mill (IKA, Labortechnik, Staufen, Germany). Two sub-samples and two assays for each sub-sample have been considered.

The content of total chlorophyll and carotenoids were determined using the spectrophotometric method described by Sumanta et al. [48], with minor modifications. Lyophilized samples (0.05 g) were mixed with 1.5 mL 80% ethanol (ethanol/water 80:20) containing 0.1% hydrochloric acid in a 10 mL screw-cap tube. The mixture underwent ultrasonic treatment using an ultrasonic power cleaner (DU-32 Digital; Argo Lab, Carpi, MO, Italy) at room temperature for 30 min. After sonication, samples were centrifuged in a refrigerated centrifuge (Beckman Coulter AllegraTM 25, Fullerton, CA, USA) for 15 min at 4000× *g* and 4 °C. The supernatant was collected, and this process was repeated twice. The combined supernatants were filtered using reinforced nylon membrane filters (0.22 μm). The filtered extract was analyzed using a spectrophotometer (Evolution 201 UV-Visible Spectrophotometers, Thermo Scientific Waltham, MA, USA) at wavelengths of 664 and 649 nm. Chlorophylls were calculated using Equations (1)–(3); Carotenoids were calculated using Equation (4).

Chl a = 13.36 (A664) − 5.19 (A649), (1)

Chl b = 27.43 (A649) − 8.12 (A664), (2)

Chl total = 17.32 (A649) + 7.18 (A664)(3)

Carotenoids = [1000 (A470) − 2.13Chl a − 97.63Chl b]/209,(4)

For anthocyanin determination, the same extract solution was used, following the method reported by Sims and Gamon [49]. Absorbance was measured at 650 nm and 525 nm using the spectrophotometer. To correct for chlorophyll interference, Equation (5) was adopted.

AA = A529 − (0.288 × A650), (5)

where AA represents the corrected anthocyanin absorbance.

Total anthocyanin content was calculated as cyanidn-3-glucoside, using the corrected absorbance and a molar absorbance coefficient for anthocyanin at 525 nm of 26,900 L mol^−1^ cm^−1^ [50].

Phenols were extracted from lyophilized samples (30 mg) using 1 mL of water/methanol (20:80 *v*/*v*) at room temperature in an ultrasonic cleaner bath (DU-32 Digital; Argo Lab, Carpi, MO, Italy) for 15 min. The mixture was then centrifuged in a refrigerated centrifuge (ThermoFisher Scientific, Waltham, MA, USA) at 14,000 rpm for 15 min at 4 °C, and the supernatant was collected. This extraction process was repeated twice, and the combined supernatants were stored at −20 °C for analysis within 24 h.

Phenolic content was determined spectrophotometrically on methanolic extracts using the Folin–Ciocalteu method: 100 mL of the extracts were diluted with 3 mL of distilled water, mixed with 0.5 mL of Folin–Ciocalteu reagent, and kept at room temperature for 5 min; then 1.0 mL of 20% Na_2_CO_3_ was added to the mixture. After 45 min at 30 °C, absorbance was read at 750 nm (Shimatzu UV-1800, Shimadzu Scientific Instruments, Columbia, MD, USA). Values were determined from a calibration curve (0–250 mg L^−1^; R2 0.998) prepared with gallic acid standard solutions using the same procedure as for the extracted sample.

Flavonoid extraction was carried out on lyophilized samples (30 mg) in water/methanol (1 mL) (20:80 *v*/*v*) at room temperature in an ultrasonic cleaner bath (DU-32 Digital; Argo Lab, Carpi, MO, Italy) for 15 min. The mixture was then centrifuged in a refrigerated centrifuge (ThermoFisher Scientific, Waltham, MA, USA) at 14,000 rpm for 15 min at 4 °C, and the supernatant was collected. The extraction was repeated twice, and the combined supernatants were stored at −20 °C for analysis within 24 h. Flavonoid content was determined using the AlCl3 method [51].

Antioxidant activity was assessed using a slightly modified version of the TEAC (Trolox Equivalent Antioxidant Activity) method described by Re et al. [52], employing the decolorization assay for both hydrophilic and lipophilic antioxidants.

The concentration of NO_3_-N was determined by ion chromatography (Dionex ICS 3000; Dionex ThermoFisher Scientific, Waltham, MA, USA) after extraction from lyophilized samples of 0.5 g with 50 mL of eluent solution (3.5 mM sodium-carbonate and 1.0 mM sodium bicarbonate) following the procedure described by Conversa et al. [53].

### 3.2. Visual Traits, Biophysical and Chemical Determination of Stored Product

Post-harvest determination encompassed weight loss, headspace gases, color, and dry matter content. These determinations were performed on fresh material, considering three sub-samples for each replicate.

Color indices and dry matter content were performed according to the methods reported in Section 3.1.1.

The headspace composition inside the bags of stored material was determined using a check-point hand-held gas analyzer (PBI Dansensor, Milan, Italy), before opening the bags. The gas analysis was conducted with a needle inserted through a septum previously fixed to the bags.

Weight loss due to total transpiration, respiration, and other factors was calculated as described in Bonasia et al. [47].

Post-harvest determination encompassed chlorophyll, nitrate, cations, phenols, flavonoids, anthocyanins, carotenoids, and antioxidant activity on the stored product, previously lyophilized, considering two sub-samples and two assays for each sub-sample, according to the methods reported in Section 3.1.2.

### 3.3. Statistical Analysis

Statistical analysis was conducted using the General Linear Model procedure of the SAS software (SAS 9.1; SAS Institute, Cary, NC, USA). Before ANOVA, Bartlett’s test was used to test the assumption that variances are equal across groups. In case of not equal variances, data were subjected to logarithm transformation. A two-way (G and T) ANOVA RCBD and 3-way (St, G and T) ANOVA RCBD was used for data processing related respectively to raw material and stored product. Mean comparisons were performed using the least significant difference (LSD) test at a significance level of *p* = 0.05.

## 4. Conclusions

The application of zeolite, as a novel elicitor, and methyl-jasmonate (MeJA), both in an unusual vegetable product (baby leaf) and/or in an unusual application mode (pre-harvest), has proven effective in improving product quality, differentially according to genotype, without compromise yield. MeJA improved quality in chicory, while zeolite improved that of kale. Notably, the combinations MeJA with chicory and zeolite with kale effectively maintained a significant quality profile in the product stored for seven days.

To elucidate the underlying mechanisms involved in genotype-elicitor interactions, further comprehensive investigations are required. These studies should aim to characterize the properties of zeolite particle films and their interactions with leaf morphological and anatomical structures. Such research will provide insights into leaf light reflectance, the generation of bioavailable forms of silicon and the associated bio-stimulant effect, particularly in relation to the activation of secondary metabolic pathways.

Foliar spray of the present elicitors could be considered innovative, in addition to safe and environmentally friendly methods for enhancing phytochemical contents of fresh material, quality and shelf life of stored products.

## Figures and Tables

**Table 1:** Effect of foliar application of elicitors on bio-physical and visual traits of kale and chicory baby leaves, at harvest.

	Fresh Yield (kg m^−2^)	Dry Matter (g kg^−1^ f.w.)	Leaf Area (cm^2^ plant^−1^)	Fresh Weight (g plant^−1^)	Leaves (n. plant^−1^)	L * (-)	h° (-)	C* (-)	Chlorophyll (µg g^−1^ f.w.)
Genotype (G)									
Kale	1.0 b ^2^	85.3 a	30.4 b	1.3 a	4.0 b	59.4 b	136.5 a	24.8 b	132.7 a
Chicory	1.2 a	73.9 b	43.6 a	1.3 a	4.8 a	70.7 a	122.4 b	33.4 a	86.1 b
Treatment (T)									
Control	1.1 a	78.6 a	35.6 a	1.3 a	4.4 a	64.3 a	131.0 a	29.4 a	101.4 a
MeJA	1.1 a	82.4 a	38.8 a	1.3 a	4.3 a	66.0 a	125.9 a	30.1 a	110.7 a
Zeolite	1.0 a	79.7 a	36.6 a	1.2 a	4.4 a	64.8 a	131.4 a	27.9 a	116.0 a
Significance ^1^									
G	*	***	**	ns	**	*	***	***	**
T	ns	ns	ns	ns	ns	ns	ns	ns	ns
G × T	ns	*	ns	ns	ns	ns	ns	ns	*

^1^ n.s., *, ** and *** not significant or significant at *p* ≤ 0.05, 0.01 and 0.001, respectively. ^2^ Means in columns not sharing the same letters are significantly different according to the LSD test (*p* = 0.05).

**Table 2:** Effect of foliar application of elicitors on headspace gases and weight losses (WL) of kale and chicory after 7 days of storage.

	O_2_ (%)	CO_2_ (%)	Weight Losses		
			Total	Respiration	Transpiration
			(g 100 g^−1^ f.w.)		
Genotype (G)					
Kale	17.9 b ^2^	2.4 a	2.3 a	0.4 a	1.9 a
Chicory	19.2 a	1.4 b	2.2 a	0.3 a	1.9 a
Treatment (T)					
Control	18.9 a	1.7 a	2.1 a	0.5 a	1.7
MeJA	18.4 a	1.9 a	2.2 a	0.3 a	1.8
Zeolite	18.3 a	2.1 a	2.4 a	0.3 a	2.2
Significance ^1^					
G	*	*	ns	ns	ns
T	ns	ns	ns	ns	ns
G × T	ns	ns	ns	ns	ns

^1^ n.s., and * not significant or significant at *p* ≤ 0.05, respectively. ^2^ Means in columns not sharing the same letters are significantly different according to the LSD test (*p* = 0.05).

**Table 3:** Effects of storage time (St), genotype (G), and foliar application of elicitors (T) and their interactions on visual quality, biometrical and nutritional traits.

	DM (g kg^−1^ f.w.)	L* (-)	h° (-)	C* (-)	Nitrate (mg kg^−1^ f.w.)	CHL ^3^ (µg g^−1^ f.w.)	TP ^3^ (mg a.g.e. 100 g^−1^ f.w.) ^4^	FLAV ^3^ (mg q.e. 100 g^−1^ f.w.) ^4^	CAR ^3^ (mg 100 g^−1^ f.w.)	ANT ^3^ (mg c.g.e. 100 g^−1^ f.w.) ^4^	HA ^3^	LA ^3^
											(µmol T.E. g^−1^ f.w) ^4^	
Storage (St)												
St0	80 a ^2^	65 b	129 a	29 b	2681 a	116 a	78 a	28 a	2.9 a	8.0 a	6.7 b	1.0 b
St7	79 b	67 a	120 b	31 a	2151 b	109 b	77 a	20 b	2.5 a	5.6 b	8.0 a	1.3 a
Significance ^1^												
St	*	***	***	**	**	*	ns	***	ns	**	*	***
St × G	ns	ns	*	ns	ns	ns	ns	ns	ns	ns	ns	ns
St × T	ns	ns	ns	ns	ns	ns	ns	ns	ns	ns	ns	ns
St × T × G	*	ns	ns	ns	ns	ns	ns	ns	ns	ns	ns	ns

^1^ n.s., *, ** and *** not significant or significant at *p* ≤ 0.05. 0.01 and 0.001, respectively. ^2^ Different letters within the column indicate significant differences at *p* = 0.05. ^3^ DM = dry matter content; CHL = total chlorophyll content; TP = total phenolic content; FLAV = total flavonoid content; CAR = total carotenoid content; ANT = total anthocyanin content; HA = hydrophilic antioxidant activity; LA = lipophilic antioxidant activity. ^4^ a.g.e = acid gallic equivalent; q.e. = quercetin equivalent; c.g.e. = cyanidin-3-glucoside equivalent. T.E. = Trolox equivalent.

## Data Availability

The data supporting the findings of this study are available from the corresponding authors upon request.

## Supplementary Materials

The following supporting information can be downloaded at: https://www.mdpi.com/article/10.3390/plants14060863/s1, Figure S1: The hue angle in kale and chicory baby leaves, at harvest (ST0) and after 7 days of storage (ST7). Vertical bars (standard error) (n = 9) with different letters are significantly different according to the LSD test (*p* = 0.05); Figure S2: Framework of the research; Table S1: Effect of foliar application of elicitors on nutritional and anti-nutritional traits of kale and chicory grown as baby leaf, at harvest.
