# Supplementary material for: Pre-Harvest Strategy for Improving Harvest and Post-Harvest Performance of Kale and Chicory Baby Leaves
Source: Plants (Basel). 2025 Mar 10;14(6):863. doi: 10.3390/plants14060863 (PMC11945244; doi:10.3390/plants14060863)
Supplement: Supplementary file 1 [file plants-14-00863-s001.zip › Figure S1 - hue.pdf]

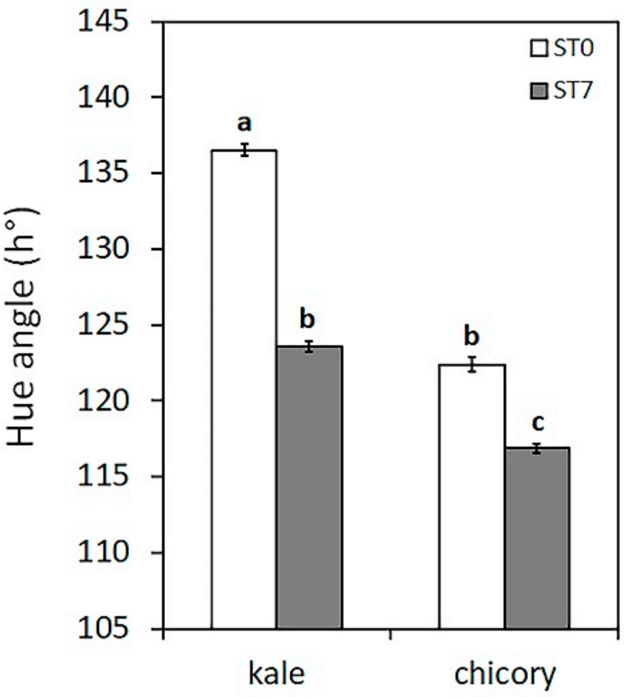

Figure S1. The hue angle in kale and chicory baby leaves, at harvest (ST0) and after 7 days of storage (ST7). Vertical bars (standard error) (n = 9) with different letters are significantly different according to the LSD test (p = 0.05).
