# Supplementary material for: Pre-Harvest Strategy for Improving Harvest and Post-Harvest Performance of Kale and Chicory Baby Leaves
Source: Plants (Basel). 2025 Mar 10;14(6):863. doi: 10.3390/plants14060863 (PMC11945244; doi:10.3390/plants14060863)
Supplement: Supplementary file 1 [file plants-14-00863-s001.zip › Table S1 - antioxidants.pdf]

Table S1. Effect of foliar application of elicitors on nutritional and anti-nutritional traits of kale and chicory grown as baby leaf, at harvest.

	Nitrate (mg kg^{-1} f.w.)	Phenols (mg a.g.e. 100 g^{-1} f.w.) [3]	Flavonoids (mg q.e. 100 g^{-1} f.w.) [3]	Carotenoids (mg 100 g^{-1} f.w.)	Anthocyanins (mg c.g.e. 100 g^{-1} f.w.) [3]	Antioxidant activity (µmol T.E. g^{-1} f.w)[3]	
						hydrophilic	lipophilic
Genotype (G)							
Kale	2,759a[2]	86.1a	29.5a	3.0a	5.2a	7.05a	1.11a
Chicory	2,604a	69.4b	26.3b	2.9a	6.1a	6.45a	0.93a
Treatment (T)							
Control	2,743a	76.6a	27.0a	2.8a	6.0a	6.13a	0.97a
MeJA	2,823a	72.9a	28.0a	3.1a	4.6a	7.09a	1.07a
Zeolite	2,478a	83.7a	28.7a	2.9a	6.3a	7.03a	1.02a
Significance[1]							
G	ns	*	*	ns	**	ns	ns
T	ns	ns	ns	ns	ns	ns	ns
G*T	*	*	*	*	*	*	*

[1]n.s., *, and ** not significant or significant at $p \leq 0.05$ and 0.01, respectively. [2]Means in columns not sharing the same letters are significantly different according to the LSD test (p=0.05). [3]a.g.e= acid gallic equivalent; q.e.= quercetin equivalent; c.g.e. = cyanidin-3-glucoside equivalent; T.E.=Trolox equivalent.
