# Supplementary figures and images for: Pre-Harvest Strategy for Improving Harvest and Post-Harvest Performance of Kale and Chicory Baby Leaves
Source: Plants (Basel). 2025 Mar 10;14(6):863. doi: 10.3390/plants14060863 (PMC11945244; doi:10.3390/plants14060863)

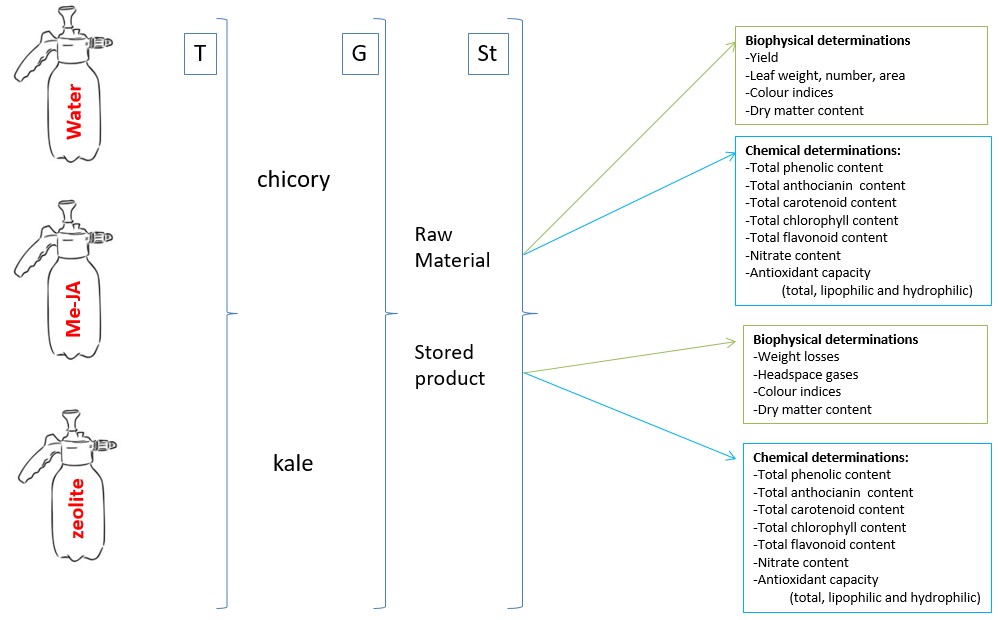

Supplement: Supplementary file 1 [file plants-14-00863-s001.zip › Figure S2 - Framework of the research NEW.jpg]
